# Smart Sensor for Online Detection of Multiple-Combined Faults in VSD-Fed Induction Motors

**DOI:** 10.3390/s120911989

**Published:** 2012-08-30

**Authors:** Armando G. Garcia-Ramirez, Roque A. Osornio-Rios, David Granados-Lieberman, Arturo Garcia-Perez, Rene J. Romero-Troncoso

**Affiliations:** 1 HSPdigital-CA Mecatronica, Facultad de Ingenieria, Universidad Autonoma de Queretaro, Campus San Juan del Rio, Rio Moctezuma 249, Col. San Cayetano, San Juan del Rio, Qro. 76807, Mexico; E-Mails: aggarcia@hspdigital.org (A.G.G.-R.); raosornio@hspdigital.org (R.A.O.-R.); granlieber@hspdigital.org (D.G.-L.); 2 HSPdigital-CA Telematica, DICIS, Universidad de Guanajuato, Carr. Salamanca-Valle km 3.5+1.8, Palo Blanco, Salamanca, Gto. 36885, Mexico; E-Mail: agarcia@hspdigital.org

**Keywords:** smart sensor, induction motors, multiple-combined faults, VSD, FPGA

## Abstract

Induction motors fed through variable speed drives (VSD) are widely used in different industrial processes. Nowadays, the industry demands the integration of smart sensors to improve the fault detection in order to reduce cost, maintenance and power consumption. Induction motors can develop one or more faults at the same time that can be produce severe damages. The combined fault identification in induction motors is a demanding task, but it has been rarely considered in spite of being a common situation, because it is difficult to identify two or more faults simultaneously. This work presents a smart sensor for online detection of simple and multiple-combined faults in induction motors fed through a VSD in a wide frequency range covering low frequencies from 3 Hz and high frequencies up to 60 Hz based on a primary sensor being a commercially available current clamp or a hall-effect sensor. The proposed smart sensor implements a methodology based on the fast Fourier transform (FFT), RMS calculation and artificial neural networks (ANN), which are processed online using digital hardware signal processing based on field programmable gate array (FPGA).

## 1. Introduction

Induction motors are widely used in industry due to their robustness, low cost, easy maintenance and versatility; representing 85% of power consumption worldwide. Thus, when induction motors start developing incipient faults [1] it is important to detect the fault early because at this stage it is easier to repair, benefiting the industry in cost and maintenance time. Faults in induction motors may produce unanticipated interruptions on production lines, with severe consequences in product quality, safety and cost. For this reason, early fault detection in induction motors has attracted the interest of many researchers in recent years [2–5]. Induction motor faults are mainly associated to bearing defects (BD), rotor faults such as broken bars (BRB) [6–18], unbalance (UNB) [19] and misalignment (MAL) [20–23]. According to these faults, sometimes two or more of them may develop simultaneously, making it important to identify if they are alone or combined. Thus far, the combined fault identification in induction motors represents a big challenge, but it has been rarely considered in spite of being a common situation, because it is difficult to identify two or more faults simultaneously online through sensors [24–31]. Besides, the connection of induction motors through variable speed drives (VSD), which allow controlling their rotational speed, extending their useful life, and saving energy is a common practice in industry [32–35], but with the undesired effect of making the detection of faults more difficult because of the spurious harmonics induced by the VSD operation.

A number of vibration and current analysis-based techniques exist for identifying specific faults in induction motors. Regrettably, most of the condition-monitoring techniques for early fault detection focus on the detection of single specific faults. Broken rotor bar condition is one of the most difficult faults to detect because the induction motor works normally without perceivable anomalies, making this fault one of the most studied in research literature. For instance, in [6] the half broken bar condition is detected by combining the correlation of the vibration and current spectra. Then, a post processing technique is applied to improve the detectability and present a motor diagnosis. In a different case, in [7] the discrete wavelet transform (DWT) is applied to the instantaneous power signal to study the case of one and three broken bars for an induction motor. Otherwise, in [8] one broken bar is detected by applying the DWT to the induction motor current at the start-up transient, and through a weighting function granting the motor diagnosis. On the other hand, after unbalance, misalignment is the second most common fault causing life reduction in induction motors. For instance, in [20] misalignment is diagnosed through unique vibration features exhibited in the full spectrum computed by the fast Fourier transform (FFT). In [22] the misalignment behavior of the rotor in an induction machine is investigated with its vibration waveforms using orbit plots and conventional FFT to identify their unique vibration features. Unfortunately, few works are related to the diagnosis and the identification of multiple combined faults. Ballal *et al.* [25] develop a combined method with artificial neural networks (ANN) and fuzzy logic to detect stator inter-turn insulation and bearing wear faults in single-phase induction motor. They take five measurable parameters (motor intake current, speed, winding temperature, bearing temperature and the noise of the machine) for the input of the adaptive neural fuzzy inference system (ANFIS) to provide a diagnosis of the induction machine. Garcia-Perez *et al.* [28] proposed a method that combines a finite impulse response (FIR) filter bank with high resolution spectral analysis based on multiple signal classification (MUSIC) for detecting multiple combined faults, analyzing vibration and current signals. The results show concordance with the analytical predetermined fault frequency for single and two or three combined faults (BRB, UNB, and BD). Romero-Troncoso *et al.* [29] performed a methodology using the information entropy and a fuzzy logic analysis in an FPGA device to identify faults like BD, UNB, BRB and their combinations by analyzing one phase of the induction motor steady-state current signal. Lebaroud *et al.* [31] presented a diagnosis method of multiple combined faults based on time-frequency classification of the current signals. All the aforementioned theoretical frameworks focus on fault detection of induction motors directly connected to the power line supply and do not consider the case of motors connected through a variable speed drive (VSD). Therefore, few works are related to faults in induction motors connected through VSDs. For instance Obaid *et al.* in [32] examined the effect of changing the input frequency in an induction motor fed through a VSD with faults such as unbalance and misalignment; the work highlighted that the harmonics induced by the VSD do not change the conditions of the frequencies of interest. Other research [34], reports a methodology for the detection of broken bars in induction motors connected through a VSD at different frequencies from 30 to 60Hz or directly to the power line supply and this methodology is based on electrical current transient analysis through DWT. The work of Cabal-Yepez *et al.* in [35] proposed the use of information entropy as a tool for multiple fault detection on induction motors controlled by a VSD obtaining results in different frequencies of operation from 30 to 50 Hz. Unfortunately, the aforementioned methodologies are based on an offline diagnosis, except for [34] that presents an online diagnosis for BRB. From an industrial point of view an online system that ensures the diagnosis of multiple-combined faults in a VSD-fed induction motor is nowadays a necessity for reducing power consumption and preventing any further damage.

From the technological point of view, smart sensors can be used to overcome the monitoring system demands due to their versatility and ability to work in environments where the access for field workers is limited, and their features in communication and data processing functionalities [36]. On the other hand, smart sensors based on field-programmable gate arrays (FPGA) are capable of performing the task due to their high-speed processing capabilities, reconfigurability, and system-on-a-chip (SoC) solutions. Smart sensors have being applied in different research areas [36–45]. For instance, Granados-Lieberman *et al.* [38] developed an FPGA-based smart sensor for real-time high-resolution frequency measurement in accordance with international standards of power quality monitoring, using a current clamp as primary sensor and the chirp z-transform (CZT) as signal processing for the diagnosis. Humin *et al.* [39] presented a smart sensor for medium-voltage dc power grid protection via current and voltage transformers. Otherwise, Rodriguez-Donate *et al.* [40] proposed a smart sensor to obtain several parameters related to motion dynamics using two primary sensors: an encoder and an accelerometer on a single link of industrial robots. In biology, Millan-Almaraz *et al.* [42] showed a smart sensor that can estimate plant transpiration. This smart sensor fuses five primary sensors: two temperature sensors, two relative humidity sensors and a light sensor. Depari *et al.* [44] presented a sensor network connected through a universal serial bus (USB)-to-Ethernet gateway for industrial applications. In [45] Son *et al.* developed a smart sensor system for machine fault diagnosis using three different sensors: vibration, current, and flux acquiring their signals, processing and diagnosing offline in a personal computer (PC). Due to their proven reliability in different research areas, smart sensors are the best suited candidates for induction motor fault monitoring systems with the presence of single or multiple-combined faults when the motor is fed through a VSD rather than having costly monitoring systems with several independent sensors and processing units connected through a computer network.

The contribution of this work is the development of a smart sensor for on-line detection of single or multiple-combined faults in induction motors connected through a VSD over a wide frequency range, covering low frequencies from 3 Hz and high frequencies up to 60 Hz, extending previously reported frequency ranges. The proposed smart sensor can use a commercially available current clamp or a Hall-effect sensor as the only primary sensor required, contrary to other works that use two or more sensors to identify a fault. The use of a current clamp as primary sensor provides additional benefits in portability, allowing one to perform the fault diagnosis in different motors without interrupting their operation. Another contribution of this work is the methodology, due to its simplicity and the theoretical foundation to analyze the frequencies of interest excited by the failure; this methodology is based on FFT fused with artificial neural networks, which is implemented into an FPGA due to its high-performance computational capabilities. In the proposed methodology, FFT provides the induction motor current spectrum normalized at steady-state, which allows covering motors with different power capabilities and a wide load range; then, specific frequency components are selected to compute their RMS to be inputs of the artificial neural network, which gives an online identification of single or combined faulty conditions. In this paper, three different faults in an induction motor: BRB, UNB, MAL and their combinations are investigated. Results confirm the potentiality of the smart sensor as an instrument for single and multiple-combined faults online detection.

## 2. Theoretical Background

### 2.1. Fault Effect on Stator Current Components

This article focuses on three different induction motor faults and their combinations: broken rotor bars (BRB), unbalance (UNB), and misalignment (MAL). The presence of BRB in induction motors produces several problems, such as power quality degradation [23]. On the other hand, UNB is the most observed fault in induction motors, and can cause catastrophic damages if not remedied. Finally, MAL is the second most commonly observed fault in rotating machines, and it is estimated to cause over 70% of the rotating machinery vibration problems [20].

#### 2.1.1. Broken Rotor Bar

**Figure 1.**
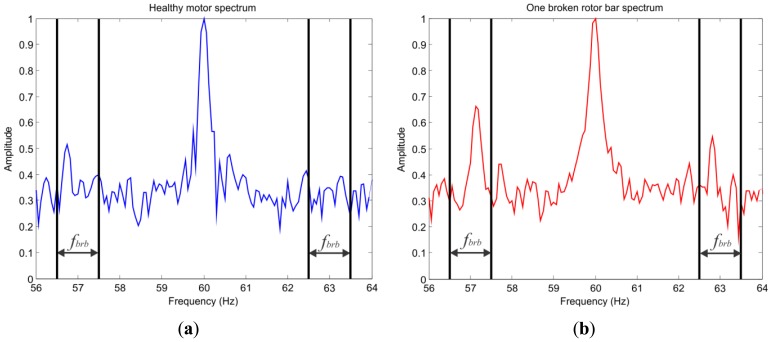
Left and right sideband components. (**a**) Healthy motor; (**b**) Broken rotor bar fault.

The detection of a broken bar fault can be done by the observation of the space harmonics *f_brb_* components in the motor current as a fault indicator:

(1) $$f_{\text{brb}}=f(1\pm 2ks)$$

where *f* is the input frequency, *k* is the harmonic index, *s* is the slip. These components are known as left sideband component and right sideband component. When a bar is broken, the amplitude of these sideband components increases significantly, as shown in Figure 1, where the markers shown delimit the sideband component area [23].

#### 2.1.2. Unbalance

The unbalance condition is presented when the mechanical load in the induction motor is not uniformly distributed, taking the center of mass out of the motor shaft. Unbalance in induction machines creates air-gap eccentricities, which change the frequency spectrum of the supply current [19].

#### 2.1.3. Misalignment

**Figure 2.**
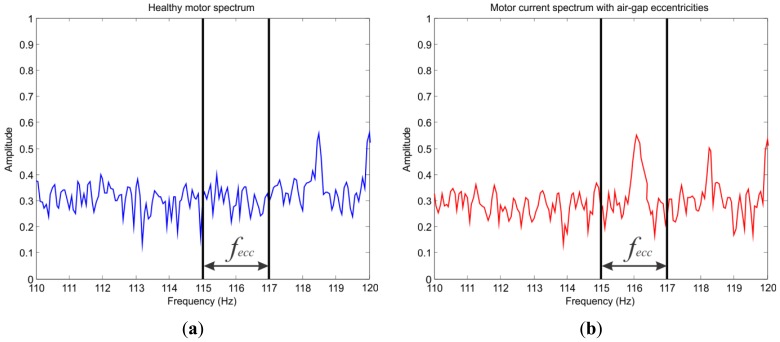
Air-gap eccentricities. (**a**) Healthy motor current spectrum; (**b**) Motor current spectrum with air-gap eccentricities.

The misalignment in induction motors occurs when the motor and the load pulleys are not aligned. The misalignment condition, like unbalance, creates air-gap eccentricities changing the frequency spectrum of the supply current [19]. The air-gap eccentricity affects the inductances of the motor resulting in harmonics (*f_ecc_*) at rotating frequency sidebands of the supply frequency predicted by Equation (2):

(2) $$f_{\text{ecc}}=f\left[1\pm k\left(\frac{1-s}{p}\right)\right]$$

where *p* is the number of pole pairs. Figure 2(a) shows the air-gap eccentricities in the healthy motor current spectrum and Figure 2(b) shows the air-gap eccentricities in a motor current spectrum with unbalance where the regions of interest are delimited.

### 2.2. Artificial Neural Networks

**Figure 3.**
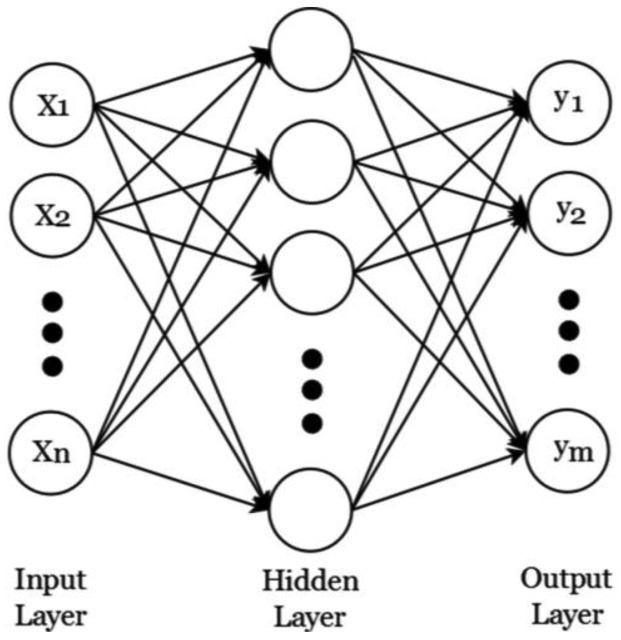
Multilayer feed-forward network architecture.

Artificial neural networks (ANN) are computational models that simulate the neurological structure of the human brain and its capability to learn and solve problems through pattern recognition. There are different ANN architectures, such as multilayer feed-forward networks (MFN), recurrent networks, feedback networks, radial basis function networks, and Kohonen self-organizing map networks. The most popular architecture for ANN is the MFN that has an input layer, an output layer and one or more hidden layers. In this ANN architecture the data moves in only one direction, from the input neurons through the hidden neurons to the output neurons, as shown in Figure 3. Where *X_i_* (*i* = *1,2,…,n*) are inputs and *y_i_* (*i* = *1,2,…,m*) are outputs. An MFN is usually trained by the back-propagation algorithm (BPA), which is a supervised learning method, and consists on mapping the process inputs to the desired outputs by minimizing the error between the desired outputs and the calculated outputs [46]. The MFN architecture is simple and practical in terms of classifier and computational load, making it an excellent candidate to be implemented in the methodology.

### 2.3. Variable Speed Drive

In industry the operation of induction motors through variable speed drives (VSDs) is very common, since it allows controlling their rotational speed, extending their useful life, and saving energy [32–35]. There are two different kinds of control in VSDs: Vector control drive and the Scalar control drive. The first one is an excellent driver to handle transients. It also enables fast control of torque speed. Some disadvantages are the complexity and the high price of the circuit. This control is commonly used in high precision tasks. As for scalar control drives, they are widely used in the industry due to their low-cost, simple design and high immunity to feedback signal errors. That type of control is preferred for simple tasks like those of pumps and fans [47,48]. When the speed varies under vector control drives the frequency content of the monitoring signals are affected by the controller bandwidth. However, it is possible to extract the condition monitoring information from signals derived within the controller [49]. For instance, in [50] rotor failures in induction motors, fed by a vector and scalar control, are diagnosed with three different signals: voltage, current and speed. In scalar control drives the characteristic harmonics of broken rotor bars in current are clearly visible and generate the same speed ripples, contrary to vector control drive where these harmonics are not affected and the speed spectrum is perfect.

## 3. Methodology

This section shows the proposed methodology for the smart sensor development, the configuration and the block diagram of the FPGA-based smart processor. First, the general structure of the smart sensor is discussed, then the smart processor architecture with the processing stages and finally, the proposed ANN.

### 3.1. Smart Sensor

**Figure 4.**
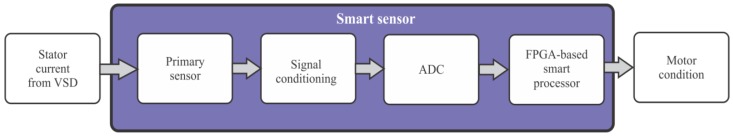
Block diagram of the fault detector smart sensor.

The block diagram of Figure 4 shows the proposed smart sensor for fault detection. The system uses a primary sensor (current clamp or Hall-effect sensor) to measure one phase of the stator current in the induction motor connected through a VSD; then, signal conditioning is applied. Subsequently, the conditioned signal is digitalized in the analog-to-digital converter block (ADC). Finally, the digital information is passed through the smart processor that is in charge to assert the motor diagnostic.

#### 3.1.1. FPGA-Based Smart Processor

**Figure 5.**
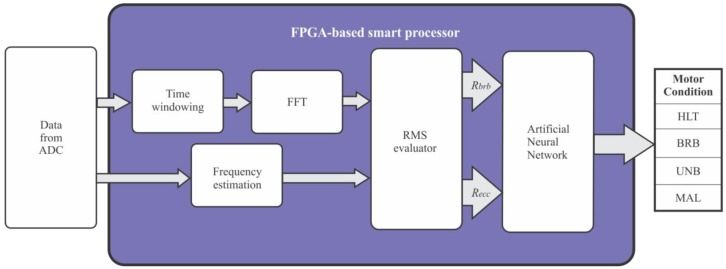
Block diagram of the FPGA-based smart processor.

**Figure 6.**
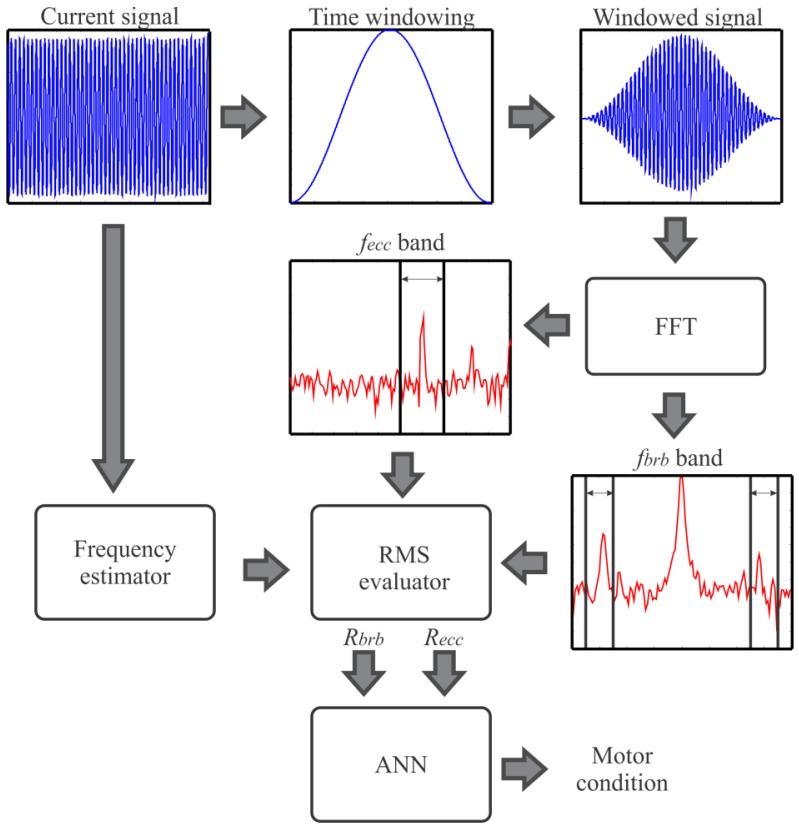
Smart processing flow up.

The block diagram of the FPGA-based smart processor internal structure to determine the condition of the motor is shown in Figure 5. The outgoing data from the ADC is time windowed with a Hanning window to reduce the leakage in the frequency domain and the frequency operation of the VSD is computed by a frequency estimator. Then, FFT is applied to get the current spectrum. In order to cover motors with different power capabilities and a wide load range the spectrum is normalized according to the magnitude of the fundamental frequency. Afterward, the bands of interest for the different faults are evaluated through the estimation of the RMS value of these selected bands according to Equations (3) and (4). The selection of the bands of interest is based on intervals between a minimum and maximum slip from 1% to 20% in order to fulfill the NEMA standard of A, B, C and D designs [51]. These slip percentages guarantee a motor load range between 25 to 100%, nevertheless lower values of this range cannot be detected. Finally, the data from each RMS evaluator are inputs of the ANN to deliver the motor condition. Figure 6 shows the smart processing flow up to detect single and multiple-combined faults in induction motors.

### 3.2. Proposed ANN

**Figure 7.**
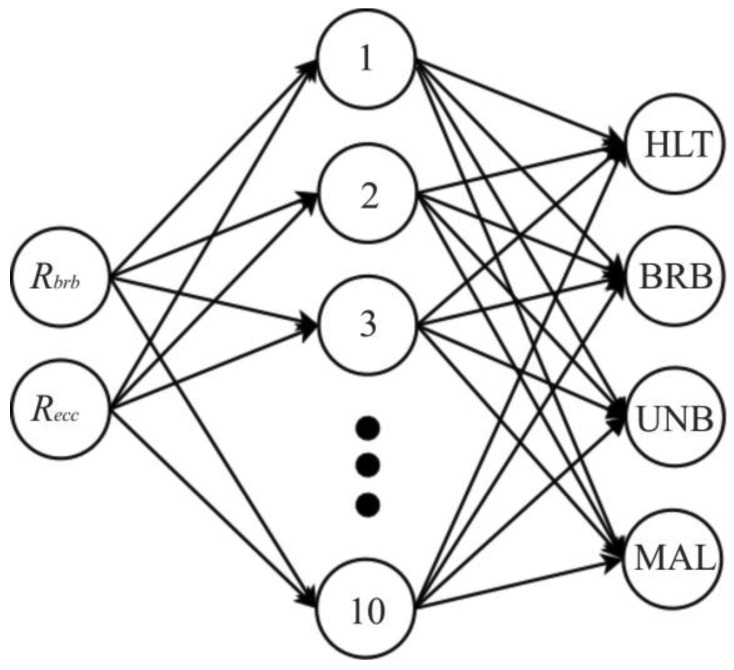
Proposed ANN.

The proposed ANN implements an MFN with two input nodes that receive the RMS value of the left and right sideband components (*R_brb_*) and the RMS value of air-gap eccentricities (*R_ecc_*) of the stator current from VSD, ten nodes in the hidden layer and four output nodes to detect: healthy motor (HLT), broken rotor bars (BRB), unbalance (UNB), misalignment (MAL) and their combinations. The output nodes correspond to each single fault condition, and if two or more faults are presented at the same time, the corresponding output nodes will be triggered up. Figure 7 shows the proposed ANN.

(5) $$R_{\text{ecc}}=\sum_{s=0.01}^{0.20}f\left[1\pm k\left(\frac{1-s}{p}\right)\right]$$

(6) $$R_{\text{brb}}=\sum_{s=0.01}^{0.20}f(1\pm 2ks)$$

## 4. Experiments and Results

In this section, the experimental setup and the results are presented for validation the proposed smart sensor. The online fault detection was performed during the steady-state of the induction motor.

### 4.1. Experimental Setup

**Figure 8.**
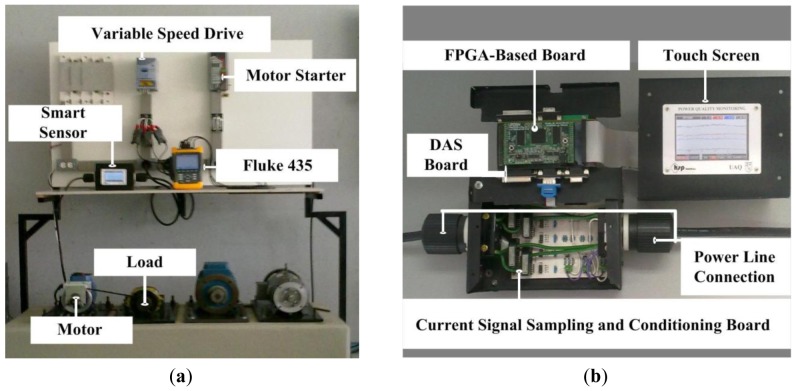
(**a**) Experiment setup; (**b**) Smart sensor.

The experimental setup consists in using the steady-state current signal provided by a VSD (model WEG CFW08) to the motor under test for detecting the multiple-combined faults and to classify the conditions of the induction motor. The VSD has an operation range from 0 Hz up to 100 Hz using a frequency resolution of 0.01 Hz. Figure 8(a) shows the experiment setup where three different 1-hp three-phase induction motors (model WEG 00136APE48T) are used for testing the performance of the proposed methodology identifying the single and multiple combined fault conditions treated in this work. The tested motors have 2 poles, 28 bars and receive a power supply of 220 V AC. The motor rotational speed is controlled through a VSD at 3 Hz, 30 Hz and 60 Hz. The applied mechanical load is of an ordinary alternator, which represents a quarter (25%) of nominal load for the motor. The current signal is acquired using a hall-effect sensor model L08P050D15, from Tamura Corporation. A 16-bit 4-channel serial-output sampling analog-to-digital converter ADS8341 from Texas Instrument Incorporated is used in the data acquisition system (DAS). The instrumentation system which was calibrated through the Fluke 435 uses a sampling frequency *f_s_* = 256 Hz obtaining 4,096 samples during 16 seconds of the induction motor steady-state and has a bandwidth of 128 Hz, which covers the VSD operation range. The motor start-up is controlled by a relay in order to automatize the test run. The acquired information is analyzed by the proposed smart sensor that is implemented in a proprietary Spartan 3E XC3S1600 FPGA platform running at 48 MHz that provides the induction motor condition as shown in Figure 8(b). Table 1 summarizes the resource usage of the FPGA.

#### 4.1.1. Single Faults

**Figure 9.**
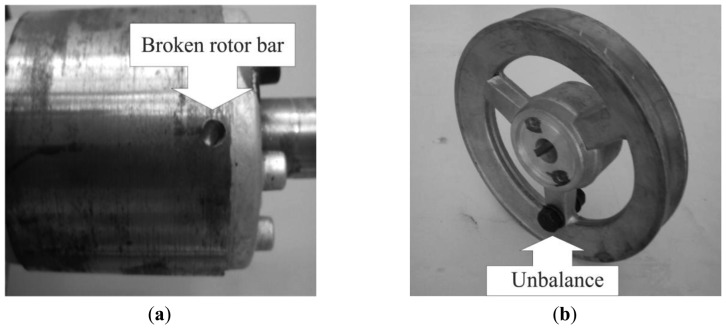
(**a**) Broken rotor bar; (**b**) Unbalance.

**Figure 10.**
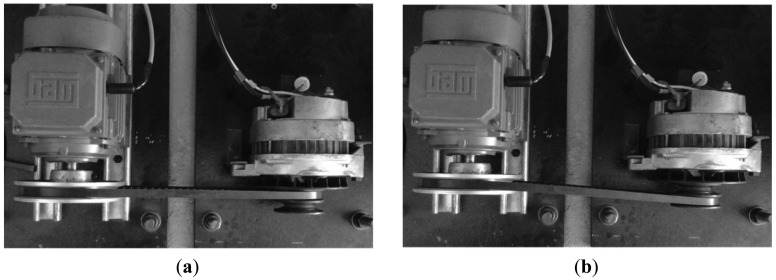
(**a**) Motor aligned; (**b**) Motor misaligned.

To produce an artificial broken rotor bar condition it was necessary to drill a 2.0 mm diameter hole in a bar of the rotor without harming the rotor shaft. Figure 9(a) shows the rotor with the broken bar used during the test. The unbalance condition was produced artificially by a bolt in the rotor pulley as shown in Figure 9(b). The misalignment test was carried out by shifting forward the band in the alternator pulley, so that the transverse axes of rotation for the motor and its load were not aligned. Figure 10(a) shows the aligned motor and the Figure 10(b) shows the misaligned motor.

#### 4.1.2. Multiple-Combined Faults

**Figure 11.**
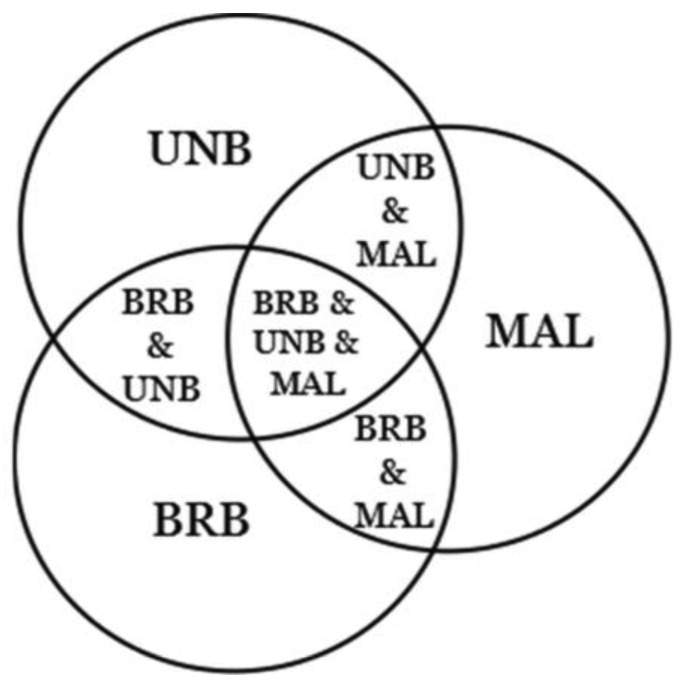
Combination for multiple-combined fault analysis.

The multiple-combined fault conditions were obtained by mixing each single fault with one or two of the remaining faults as shown in Figure 11.

#### 4.1.3. Network Training

The ANN is trained with the back-propagation algorithm to identify single or multiple-combined faults in induction motors. Forty trials are carried out under each motor condition for each study frequency. The training set was obtained with 1,000 random synthetic values for each study frequency within the range [*μ* – *σ*, *μ* + *σ*], where *μ* is the mean and *σ* the standard deviation of the RMS values of spectral components of interest from steady-state of the induction motor on the first five trials. Real values of each fault were used as validation set for the diagnosis. The weights and the biases of each layer in the ANN were obtained offline, using the Matlab neural network toolbox for being implemented on the FPGA for the online diagnosis. The ANN is trained for motors with B NEMA design, since those are used in general applications [51]. The theoretical background shows that the frequency components of the faults do not depend of the power motor capability. Nevertheless, the current magnitude depends of the load and the motor power. So as to minimize those undesired effects that could modify the ANN output the spectrum is normalized before being applied to the ANN. This guarantees the same results in motors with similar characteristics. However, other NEMA designs have different relationship between the fundamental frequency and the fault components because of changes in the current flux density and the stator field [5]. In some of these cases a new training is required for adjusting the calibration of the smart sensor and improving the classification results.

### 4.2. Fault Identification Results

Table 2 presents the results delivered by the proposed smart sensor during the induction motor condition identification for each frequency studied in order to show the effectiveness of the system. The results include the identification of a healthy condition, a single isolated fault, and the combination of two or three faulty conditions for each study frequency. In order to obtain statistically significant results, 40 tests were performed to acquire the current signals from the induction motor in all treated cases for each study frequency.

### 4.3. Discussion

Three different frequency cases are studied in order to fulfill a range from low to high frequencies: 3 Hz, 30 Hz and 60 Hz. Results of the smart sensor with the motor running at 3 Hz show an effectiveness of 100% in health motor (HLT), unbalance (UNB), misalignment (MAL), the combination of unbalance and misalignment (UNB-MAL) and the combination of broken bars with unbalance and misalignment (BRB-UNB-MAL); the results for broken rotor bars (BRB), broken rotor bars in combination of unbalance (BRB-UNB) and broken rotor bars combined with misalignment (BRB-MAL) present an effectiveness over 80%. Due to the fact the sideband frequencies at 3 Hz were closer to the fundamental frequency, the conditions with broken bars were more difficult to diagnose. On the other hand, with the motor running at 30 Hz the smart sensor shows an effectiveness of 100% with the exception of BRB-UNB-MAL which presented an 80% effectiveness. Finally, at 60 Hz of the VSD, the smart sensor presents an effectiveness of 100% with the exception of HLT and BRB-UNB-MAL with effectiveness over 80%. A significant characteristic of the proposed smart sensor is the detection of single and multiple-combined faults in VSD-fed induction motors in an automatic way with only a primary sensor, different from the reviewed literature where the results are from single faults or multiple-combined faults interpreted offline by the user from current of the power supply or vibration signals of the induction motor with two or more primary sensors. Table 3 shows the faults detected by the proposed smart sensor (PSS), and the works that fulfill some of the faults detected and their combination. For instance, in [29,30] show online methodologies for the detection of multiple-combined faults of induction motors connected through the power line supply. On the other hand, in [32], reports an offline methodology for the detection of UNB and MAL at different operation frequencies. In a different case, [35] presents a methodology for the detection of different single isolated faults at different operation frequencies over 30 Hz. The PSS offers an online detection of single and multiple-combined faults at different operation frequencies in a wide range from 3 Hz to 60 Hz.

## 5. Conclusions

This work proposes a new smart sensor for online detection of multiple-combined faults in VSD-fed induction motors using only a Hall-effect current sensor as primary sensor in one-phase of the induction motor, which results in a high portability. The proposed methodology is based on the FFT and an ANN classifier in order to determine the motor condition according to the motor operation frequency controlled by the VSD, the simplicity of this methodology allows analyzing the frequencies of interest excited by the different failures. The FFT spectrum is normalized in order to cover different power motor capabilities and a wide load range. The functionality of the smart sensor was successfully tested in forty tests of each category of the faults and their combinations. Results demonstrate that the proposed smart sensor is highly efficient in effecting a diagnosis of the induction motor operating over a wide frequency range of the VSD (3, 30 and 60 Hz), different from other works [7–31] that show results from motors fed by the power line supply only or [32–34] that present results from VSD-fed ones, but in a narrow frequency range without combining faults. The obtained results show the versatility of the proposed smart sensor for its use in diverse industrial applications that employ induction motors fed by a VSD. The proposed smart sensor allows the early fault detection benefiting the industry in cost and maintenance time. The proposed smart sensor for online detection of multiple-combined faults in VSD-fed induction motors is based on FPGA technology that provides high computation performance for the proposed methodology, as well as a low-cost, portable and efficient solution. This implementation shows that an FPGA platform is a suitable solution for smart processing units in developing smart sensors.

## Figures and Tables

**Table 1.** Resource usage of the FPGA.

**Resource utilization**	**Xilinx Spartan 3E XC3S1600E**
**Slices**	1,757/14,752 (12%)
**Flip-flops**	638/29,504 (2%)
**4-input LUTs**	3,270/29,504 (11%)
**Maximum operation frequency**	53.012 MHz

**Table 2.** Effectiveness of the proposed smart sensor on identifying the induction motor condition with one or multiple combined faults.

**Induction motor condition**	**03 Hz Effectiveness (%)**	**30 Hz Effectiveness (%)**	**60 Hz Effectiveness (%)**
**HLT**	100	100	80
**BRB**	80	100	100
**UNB**	100	100	100
**MAL**	100	100	100
**BRB-UNB**	80	100	100
**BRB-MAL**	90	100	100
**UNB-MAL**	100	100	100
**BRB-UNB-MAL**	100	80	90

**Table 3.** Comparison between proposed smart sensor (PSS) and reviewed literature.

**Induction motor condition**	**VSD-fed**	**Power line supply**
**BRB**	[34], PSS	[7–19,27]
**UNB**	[32,33,35], PSS	[20]
**MAL**	[32,35], PSS	[21–23]
**BRB-UNB**	PSS	[28–31]
**BRB-MAL**	PSS	[24]
**UNB-MAL**	PSS	
**BRB-UNB-MAL**	PSS	
